# Annotations

**Published:** 1905-06-17

**Authors:** 


### ANNOTATIONS.

### The Milk Supply and Infant Feeding.

In his report on the prevention of infantile mortality, Dr. J. F. J. Sykes, the medical officer of health for St. Pancras, states some wholesome truths in vigorous terms. Recognising the necessity for encouraging the practice of breast-feeding, he proposes a series of practical measures by which such encouragement may be afforded. At the same time he advises the adoption of a policy calculated to render possible a supply of pure milk for those infants who, under the compulsion of circumstances, must necessarily be hand-fed. The key note of that policy is found in the phrase "complete control over the production at the farm." This is urged as the initial step, and as an essential preliminary to the establishment of any depot or place for the distribution of milk for the use of hand-fed sucklings. Such distribution, apart from supervision of the source of supply, is condemned by Dr. Sykes as absolutely wrong in principle, and milk supplied under these conditions ought, he contends, to be labelled "not for infants." Further, the report expresses a confident opinion that if a London municipal authority had the courage to establish a dairy farm in the country with a view to the production and distribution, under medical supervision, of pure milk for feeding sucklings deprived of human milk, the benefits would extend to other children and to adults, for such a step would stimulate the dairy farmers to improve the present crude methods of collection and distribution. There can be no doubt that in all this there is sense as well as science. To organise depots as channels of supply and to leave the sources of supply without control, is to institute reform at the wrong end of the process. There is a plausible appearance about such a proposal which may deceive the unwary, and attractive terms like "humanised milk" make in the same direction. As Dr. Sykes rightly says, "the only method of humanising cow's milk is by passing it through the human mother." More straight speaking of this order is much to be desired. It may help to remove from the question of infant feeding the atmosphere of names and sentiment which obscure the true facts of the case.

### Medical Defence Societies.

The recent annual meetings of the two societies specially concerned with the protection of members of the medical profession in the discharge of their important responsibilities afford convincing evidence of the valuable and necessary work which such organisations undertake. To take but a single instance: there will be fresh in the memory of our readers the case of a member of the profession who was recently cited as co-respondent in an action for divorce. The difficulties of the case were considerable, and though the absolute innocence of the incriminated practitioner was of course the main strength of the defence the establishment of this in open court was a practical testimonial to the efficiency with which the case was arranged and conducted. The risks of charges of this order are further illustrated by the statement in the report of the Medical Defence Union that during 1904 the solicitor to the Union had to deal with no fewer than 15 cases in which slanders had been circulated relative to the professional conduct of members towards female patients. It is highly satisfactory to learn that in every one of the instances the professional honour and reputation of the practitioner concerned were completely vindicated. There are of course many other directions in which the influence and value of the defence societies are expressed, and not the least of these is the expert and experienced advice they are able to offer to their members in times of difficulty. The importance of joining one of these organisations at the very outset of professional life ought to be impressed on the junior members of the profession and this advice might well be part of the counsel offered by medical schools to their students.

### The Time for Examination.

By the victims who must submit to the process, the time of year, whatever it be, selected for the conduct of an examination will probably always seem an unfortunate one. But even from their point of view one date may be more unfortunate than others, and by those who are free to consider the question in an impartial spirit and as a factor in the educational problem, the date at which examinations are suggested will be seen to be not unimportant. Assuming that the medical student is to achieve the success of a diploma on the termination of his five years' curriculum, he must pass his examinations, as a rule, either at the beginning or the end of a winter session. Some arrangements adopted by certain of the diploma-granting bodies have in comparatively recent years tended to encourage students to enter for examination in the spring rather than in the autumn months, and this, so it seems to us, is a doubtful policy. If the examination takes place in March or April, the preceding session is occupied in attendance on classes which either do or do not include the subjects embraced by the examination. In the former event the student may possibly have covered these subjects, but he has had no time for the process of mental digestion, and not unnaturally what has been acquired in these circumstances has no element of permanence. If, on the other hand, the subjects of the winter session do not coincide with those of the rapidly following examination, attendance at classes becomes little more than a formality, for attention to two rival lines of study at one and the same time is impossible. All these advantages disappear if the examination is arranged in September or October. The student may give his undivided efforts to class and hospital work during the session and be able during the long vacation to revise and assimilate in peace and quietness the knowledge he has acquired. It may be said that to fix examinations for October is to spoil the summer holiday. But the vacations, we hold, afford abundant time both for holidays and for revision work. Indeed, a further advantage of the plan here advocated is that it discourages the idea that a student of medicine ought to take two or three months' holiday every year. If he is pursuing his profession in a serious spirit he ought not to waste time in any such fashion.

